# Hyperglycemia-Induced Hypovolemia Is Involved in Early Cardiac Magnetic Resonance Alterations in Streptozotocin-Induced Diabetic Mice: A Comparison with Furosemide-Induced Hypovolemia

**DOI:** 10.1371/journal.pone.0149808

**Published:** 2016-02-22

**Authors:** Michael Joubert, Dimitri Bellevre, Damien Legallois, Nicolas Elie, Laurent Coulbault, Stéphane Allouche, Alain Manrique

**Affiliations:** 1 Diabetes care unit, Caen University Hospital, Caen, France; 2 Nuclear Medicine department, Caen University Hospital, Caen, France; 3 Cardiology unit, Caen University Hospital, Caen, France; 4 CMABIO-HIQ facility, SF4206 ICORE, IBFA, Université Caen Normandie, Caen, France; 5 Biochemical unit, Caen University Hospital, Caen, France; 6 EA4650 Université Caen Normandie, GIP Cyceron, Caen, France; Stellenbosch University, SOUTH AFRICA

## Abstract

**Aims:**

The aim of the study was to assess the early features of diabetic cardiomyopathy using cardiac magnetic resonance within the first week after streptozotocin injection in mice. We focused on the relationship between left ventricular function and hypovolemia markers in diabetic animals compared to a hypovolemic rodent model.

**Methods and Results:**

Swiss mice were randomized into control (group C), streptozotocin-induced diabetes (group D) and furosemide-induced hypovolemia (group F) groups. Cardiac magnetic resonance, non-invasive blood pressure, urine volume, plasma markers of dehydration and cardiac histology were assessed in all groups. Mean blood glucose was higher in diabetic animals than in groups C and F (30.5±5.8 compared to 10.4±2.1 and 11.1±2.8 mmol/L, respectively; p<0.01). Diuresis was increased in animals from group D and F compared to C (14650±11499 and 1533±540 compared to 192±111 μL/24 h; p<0.05). End diastolic and end systolic volumes were lower in group D than in group C at week 1 (1.52±0.36 vs. 1.93±0.35 and 0.54±0.22 vs. 0.75±0.18 mL/kg, p<0.05). These left ventricular volume values in group D were comparable to those observed in the acute hypovolemia model (group F). Increased dehydration plasma markers and an absence of obvious intrinsic myocardial damage (evaluated by cardiac magnetic resonance and histology) suggest that a hemodynamic mechanism underlies the very early drop in left ventricular volumes in group D and provides a potential link to hyperglycemic osmotic diuresis.

**Conclusions:**

Researchers using cardiac magnetic resonance in hyperglycemic rodent models should be aware of this hemodynamic mechanism, which may partially explain modifications in cardiac parameters in addition to diabetic myocardial damage.

## Introduction

Diabetes mellitus has reached epidemic proportions worldwide, and it is one of the leading causes of cardiovascular diseases, especially coronary artery disease [1,2]. It is well established that glycemic control and the optimized treatment of all other cardiovascular risk factors prevent atherosclerotic vascular events [3]. However, the presence of diabetes predicts poor prognosis in heart failure patients [4–6]. The concept of diabetic cardiomyopathy (DCM) is controversial, but accumulating data are progressively clarifying this subject [7]. The diagnosis of DCM relies on (i) the detection of structural/functional myocardial abnormalities in diabetic patients and (ii) the exclusion of other contributors to cardiomyopathy [8]. Myocardial fibrosis is frequently observed in DCM, but it cannot be used for patient management because of the invasiveness of myocardial biopsies [9]. Therefore, the diagnosis of diabetic cardiomyopathy is based on non-invasive cardiac imaging using echocardiography or cardiac magnetic resonance (CMR). Regardless of the imaging technique used, the primary findings are cardiac hypertrophy and left ventricular diastolic dysfunction with or without systolic function impairment [8]. The widespread use of Doppler tissue imaging and myocardial strain analysis using ultrasound or tagged CMR increased the early detection of subclinical cardiac dysfunction in patients with diabetes [10–12]. The pathophysiological issues of DCM are not completely understood, and these issues are being actively investigated using preclinical rodent models of diabetes. Streptozotocin (STZ)-induced diabetes is one of the most widely investigated models, and this model exhibits a diastolic/systolic dysfunction that increases in severity in proportion to diabetes duration [13]. Of note, this model allows the control of diabetes onset. However, it is unknown whether this acute onset hyperglycemia causes changes in loading conditions and thus affects cardiac function assessment.

This model was chosen to assess the early features of DCM using CMR within the first week after STZ injection and to investigate the relationship between left ventricular function and the markers of hypovolemia in diabetic animals compared to a hypovolemic rodent model.

## Methods

### Animals

All animal procedures were performed in accordance to the guidelines from Directive 2010/63/EU of the European Parliament on the protection of animals used for scientific purposes and specific French laws were followed. All investigations and procedures were approved by our regional animal ethics committee (Cenomexa 054 –N°03854.01) and all efforts were made to minimize animal suffering. In particular, animals who exhibited signs of severe illness were killed before study endpoint in order to avoid prolonged suffering. The reporting of this animal research follows the ARRIVE guidelines [14]. Experiments were conducted on 18- to 22-week-old male Swiss mice weighing 30–45 g at baseline (Janvier Labs—France). Mice were housed individually in a temperature-controlled room with ad libitum access to standard mice chow and water. Mice were first randomized into a control group (C) and a diabetes group (D). Mice from group D received 180 mg/kg of intraperitoneal (IP) STZ (Sigma-Aldrich, St. Louis, MO, USA). Mice from group C received vehicle only. Minimal insulin treatment was introduced 48 h after STZ injection in group D only: Glargine (Sanofi, Paris, France) 1 IU/day subcutaneously (SC) at 09:00 am.

Subsequently, 18- to 22-week-old male Swiss mice weighing 30–45 g at baseline (Janvier Labs, France) received two 0.4 mL successive subcutaneous injections of furosemide (250 mg/mL) separated by 2 h. Mice were denied access to water after the second furosemide injection to induce hypovolemia until cardiac function assessment [15]. These furosemide-induced hypovolemic mice were defined as group F.

### Cardiac magnetic resonance (CMR)

CMR was performed at week 1 (w1) and week 4 (w4) after IP vehicle/STZ injection in the mice from groups C/D and 4 h after the second SC furosemide injection in the mice from group F. A 7-Tesla Bruker Pharmascan magnetic resonance system (Bruker Biospin, Ettlingen, Germany) interfaced with a dedicated small-animal electrocardiographic and respiratory triggering system (SA Instruments) was used. Anesthesia was induced and maintained using isoflurane (1.5–3%) in an oxygen/nitrous oxide mix (0.6 L/min) via spontaneous breathing. The isoflurane flow was titrated to maintain a 40–60 breaths/min respiratory rate. Body temperature was maintained in a physiological range using a heating pad. Left ventricular function was assessed using a triggered cine Flash sequence (IntraGate^®^, Bruker Biospin). Six short-axis uniformly distributed slices from the base to the apex were acquired (slice thickness: 0.563 mm; Time of Echo (TE): 2 ms / Time of Repetition (TR): 50 ms; flip angle: 25°; field of view: 28x28 mm; matrix size: 128x128; and spatial resolution: 0.219x0.219 mm/pixel), with a temporal resolution of 16 images per cardiac cycle. Additional sequences were performed in mice from groups C and D for the assessment of the T2 relaxation times and myocardial strain. A multi-slice multi-echo T2 sequence using the same geometry (8 echo times, ranging from 4.77 to 38.17 ms) was used for the assessment of the T2 relaxation times, a marker of myocardial fibrosis. Myocardial strain, which is related to intrinsic deformation, was assessed using 3 short-axis and 1 long-axis tagged CMR obtained using a cine 2D Flash sequence with dual ECG and respiratory gating (slice thickness: 1 mm; TE/TR: 3/8 ms; flip angle: 15°; field of view: 45x45 mm; matrix size: 256x256; and spatial resolution: 0.176x0.176 mm/pixel).

### Cardiac function analysis

Left ventricular parameters were analyzed blindly using Segment software after manual delineation of the endocardial and epicardial borders of all slices at each time frame (Segment v1, 8 R1675, Medviso AB, University of Lund, Sweden). Left ventricular parameters were indexed to body weight if relevant: end diastolic volume (EDV—mL/kg); end systolic volume (ESV—mL/kg); stroke volume (SV—mL/kg); left ventricular mass (LVM—g/kg); ejection fraction (EF–%); cardiac output (CO—mL kg^-1^ min^-1^); power-to-mass index (CO/LVM—mL g^-1^ min^-1^); peak filling rate (PFR– μL/s); end diastolic and end systolic wall thickness (EDWT and ESWT—mm); and wall thickening fraction (WT–%). Further CMR analyses were performed using the open source software OsiriX (http://www.osirix-viewer.com/) and the InTag plugin for circumferential (Ecc), radial (Err) and longitudinal (Ell) strain assessments. The T2 fit map plugin was used for T2 assessment.

### Physiological measurements

Non-anesthetized, warmed (37°C) mice were trained 3 days in restrainers for tail-cuff inflation before the systolic blood pressure (SBP) and heart rate (HR) were recorded daily for 2 days (20 measurements in a row) using a BP-2000 blood pressure analysis system and its connected software (Bioseb, Vitrolles, France). These 2-day recordings were performed twice for each animal: at baseline and w1 in animals from groups C and D and at baseline and 4 h after the second furosemide injection in animals from group F. HR and SBP are expressed in beats per minute (bpm) and mmHg, respectively. In addition, mice from group C, D and F were also placed in metabolic cages to record the urine volume in a 24 h period.

### Blood analysis

Blood samples were collected at w1 in groups C and D and 4 h after the second furosemide injection in group F, in anesthetized animals specifically set aside for blood analysis, using direct cardiac puncture just before euthanasia. Plasma urea and creatinine concentrations were determined using a DxC system (Beckman Coulter, Paris, France). Creatinine was quantified using a modified kinetic Jaffe reaction, which was calibrated using an isotope dilution mass spectrometry-traceable reference material. The plasma urea concentration was determined using enzymatic conductivity. Plasma osmolality was measured using the freezing point depression method in a Fiske micro-sample osmometer (Advanced Instruments, Norwood, USA). Plasma aldosterone concentrations were measured using radioimmunoassay (Beckman Coulter Immunotech, France).

### Histology

Mice were euthanized by cervical dislocation under isoflurane anesthesia, and the hearts were excised and sectioned into two parts (basal and apical). Sections were fixed and embedded in paraffin. A 6 μm-section of each part was sliced and stained with Picro-Sirius Red (Ral-diagnostics, Bordeaux, France) to reveal collagen I and III fibers. Whole-slide images of histological sections were digitized at 20× using a ScanScope CS microscope slide scanner (Leica Biosystems, Nussloch, Germany). ImageScope software (Leica Biosystems, Nussloch, Germany) was used to draw global regions of interest (ROIs) for each slice. ROIs were processed to determine the amount of collagen using the programming language Python (Python Software Foundation) and the libraries Openslide, Geospatial Data Abstraction and Mahotas [16]. The amount of collagen in the basal and apical slices was averaged for analysis.

### Statistical analysis

Statistical analyses were performed using Prism 6.0f software (GraphPad Software Inc., La Jolla, CA, USA). All data are given in means±SD. Intra- and inter-group comparisons were performed using a two-tailed *t*-test or ANOVA, when appropriate. The relationship between quantitative variables was assessed using Pearson’s correlation coefficient. A p-value <0.05 denoted significance. Individual data are available in S1 Table.

## Results

### Body weight and blood glucose

Fifty animals were randomized in this study: group C, n = 18; group D, n = 20; and group F, n = 12. Baseline body weights are presented in Table 1. The body weight increased from w1 to w4 in group C (41±7 to 44±9 g; p<0.05), but it decreased in group D (37±3 to 34±4 g; p<0.05). The body weight in group F also decreased from baseline to 4 h after the second furosemide injection (33±1 to 31±1 g; p<0.05). The mean blood glucose was higher in diabetic animals than in groups C and F (30.5±5.8 compared to 10.4±2.1 and 11.1±2.8 mmol/L, respectively; p<0.01) (Table 1).

**Table 1:** Clinical and biological results of mice from groups C, D and F.

	Group C	Group D	Group F
**Body weight (g)**			
baseline	41±7	36±3*	33±1**
H+4^#^	NA	NA	31±1
week 1	41±7	37±3	NA
week 4	44±9	34±4**	NA
**Mean blood glucose (mmol/L)**	10.4±2.1	30.5±5.8****	11.1±2.8
**Creatinine (μmol/L)**	19.5±5.4	21.4±7.5	33.0±3.9^§^
**Urea (mmol/L)**	4.6±0.7	6.2±1.5	31.3±4.2^§^
**Osmolality (mosm/kg)**	306±13	346±15****	347±23**
**Aldosteronemia (pmol/L)**	71±51	846±391***	4687±1386***

Biological results of mice from groups C, D and F. Blood samples were collected at week 1 (w1) for mice from groups C and D and 4 h after the second furosemide injection for mice from group F. All values are means±SD.

^#^body weight 4 h after the second furosemide injection for mice from group F only.

^§^p<0.05 compared to groups C and D;

*p<0.05,

**p<0.01,

***p<0.001,

****p<0.0001 compared to group C.

### CMR assessment in diabetic and control animals

Twenty-two mice were explored using CMR (group C, n = 12; group D, n = 10) (Table 2). Left ventricular volumes were lower in group D compared to group C at w1 (EDV: 1.52±0.36 vs. 1.93±0.35 mL/kg (p<0.01); ESV: 0.54±0.22 vs. 0.75±0.18 mL/kg (p<0.05); SV: 0.98±0.17 vs. 1.18±0.19 mL/kg (p<0.05)). As shown in Table 2, significant differences were also observed for EDV, ESV and SV at w4. The power-to-mass ratio CO/LVM was lower in group D compared to C at w4 (215±48 vs. 295±47 mL g^-1^ min^-1^, p<0.01). Early diastolic dysfunction was found in diabetic animals compared to controls, as demonstrated by a significant decrease in PFR at w1 and w4 (Table 2). The myocardial strain (Ecc, Err and Ell) and T2 values were not different between groups at any time point.

**Table 2:** CMR results at w1 and w4 in mice from groups C (control) and D (diabetes).

		Group C (n = 12)	Group D (n = 10)
**LVM (g/kg)**	w1	2.32±0.35	2.26±0.24
	w4	2.23±0.33	2.41±0.40
**EDV (mL/kg)**	w1	1.93±0.35	1.52±0.36**
	w4	2.06±0.29	1.64±0.28**
**ESV (mL/kg)**	w1	0.75±0.18	0.54±0.22*
	w4	0.78±0.19	0.58±0.14*
**SV (mL/kg)**	w1	1.18±0.19	0.98±0.17*
	w4	1.26±0.15	1.06±0.17**
**EDWT (mm)**	w1	0.87±0.09	0.90±0.15
	w4	0.90±0.13	0.95±0.15
**ESWT (mm)**	w1	1.27±0.13	1.29±0.20
	w4	1.25±0.13	1.35±0.18
**WT (%)**	w1	46±9	43±13
	w4	39±14	42±11
**EF (%)**	w1	61.4±3.6	65.5±6.3
	w4	62.2±4.7	64.8±4.6
**CO (mL kg**^**-1**^ **min**^**-1**^**)**	w1	602±109	490±116
	w4	637±69	544±102
**CO/LVM (mL g**^**-1**^ **min**^**-1**^**)**	w1	272±66	223±65
	w4	295±47	215±48**
**PFR (μL/s)**	w1	1642±396	1296±369*
	w4	1879±455	1232±270**
**Ecc**	w1	-0.10±0.02	-0.10±0.03
	w4	-0.09±0.02	-0.10±0.01
**Err**	w1	0.07±0.01	0.07±0.02
	w4	0.06±0.02	0.08±0.02
**Ell**	w1	-0.08±0.02	-0.08±0.02
	w4	-0.08±0.02	-0.09±0.02
**T2 (ms)**	w1	26.2±4.4	25.5±4.8
	w4	24.9±2.6	26.4±3.7

CMR results at week 1 (w1) and week 4 (w4) in mice from groups C (control) and D (diabetes). All values are means±SD. LVM (left ventricular volume); EDV (end diastolic volume); ESV (end systolic volume); SV (stroke volume); EDWT (end diastolic wall thickness); ESWT (end systolic wall thickness); WT (wall thickening fraction); EF (ejection fraction); CO (cardiac output); CO/LVM (power-to-mass index); PFR (peak filling rate); Ecc (circumferential strain); Err (radial strain); Ell (longitudinal strain); T2 (T2 multi-echo sequences).

*p<0.05,

**p<0.01 compared to group C.

### CMR assessments in furosemide-treated animals

**Fig 1:**
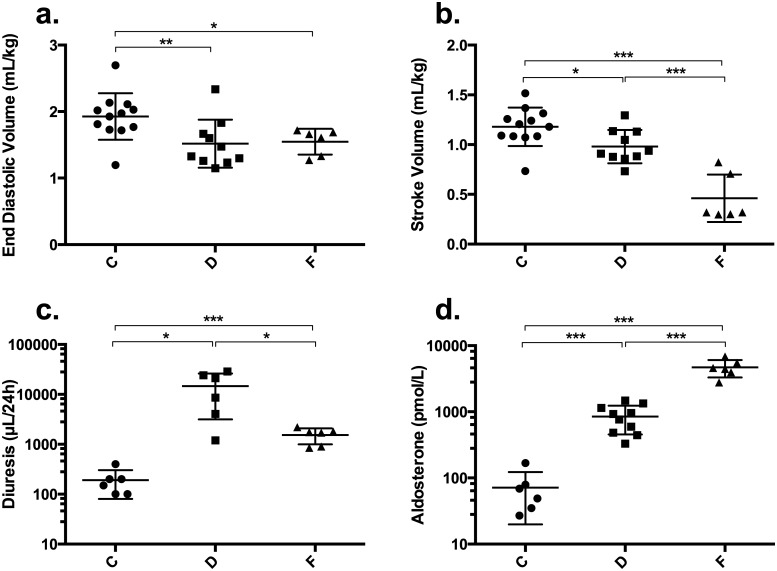
All panels: Individual data, mean (thick bar) and SD (thin bar) are presented for group C (black circles), group D (black squares) and group F (black triangles). Panels a. and b.: End diastolic volume (EDV) and stroke volume (SV) (mL/kg) at week 1 (w1) in mice from groups C (control) and D (diabetes) and 4 h after the second furosemide injection in mice from group F (furosemide-induced hypovolemia). Panel c.: diuresis (μL/24 h) in animals from groups C, D and F (log-10 scale for Y axis). Panel d.: plasma aldosterone (pmol/L) measured at w1 in mice from groups C and D and 4 h after the second furosemide injection in mice from group F (log-10 scale for Y axis). *p<0.05; **p<0.01; ***p<0.001.

Six additional mice from group F were evaluated using CMR at baseline and 4 h after the second furosemide injection. ESV remained stable (1.27±0.23 to 1.09±0.43 mL/Kg) but EF, PFR, EDV and SV decreased significantly between these two time-points from 50.2±9.6 to 31.9±20.9%, 1472±392 to 509±202 μL/s, 2.56±0.24 to 1.55±0.20 mL/kg and 1.30±0.32 to 0.46±0.24 mL/kg, respectively (p<0.05). The decrease in EDV and SV after furosemide injection in group F produced values that were similar to those of group D at w1 (Fig 1a and 1b).

### Physiological measurements

Six animals from group C, 10 animals from group D and 6 animals from group F were non-invasively explored using a tail cuff to assess HR and SBP. HR/SBP remained stable from baseline to w1 in control and diabetic animals (487±74 / 112±9 to 521±93 / 115±6 and 517±120 / 115±5 to 480±132 bpm / 114±25 mmHg, respectively). HR also remained stable from baseline to 4 h after the second furosemide injection in animals from group F (544±58 to 473±88 bpm), but SBP decreased from 125±10 to 105±9 mmHg (p<0.05).

**Fig 2:**
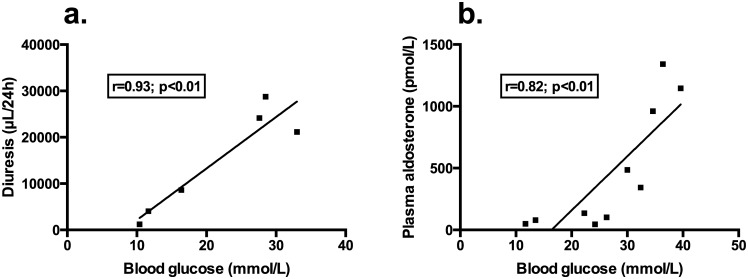
Correlation between diuresis (μL/24 h) (panel a.), plasma aldosterone (pmol/L) (panel b.) and blood glucose (mmol/L) in mice from group D.

Urine volume was assessed in 6 animals from each group: diuresis was increased in animals from group D and F compared to C (14650±11499 and 1533±540 compared to 192±111 μL/24 h; p<0.05) (Fig 1c). In addition, in group D, blood glucose positively correlated with diuresis (r = 0.93, p<0.01) (Fig 2a).

### Blood analysis

Blood samples were collected in 6, 10 and 6 animals from groups C, D and F, respectively. Plasma creatinine and urea were higher in group F than in groups C and D. Osmolality and plasma aldosterone were increased in groups D and F compared to group C (osmolality/aldosterone: 346±15 / 846±391 and 347±23 / 4687±1386 compared to 306±13 mosm/kg / 71±51 pmol/L, respectively; p<0.05) (Fig 1d). Table 1 summarizes the blood analysis results. Blood glucose positively correlated with plasma aldosterone in group D (r = 0.88, p<0.01) (Fig 2b).

### Histology

**Fig 3:**
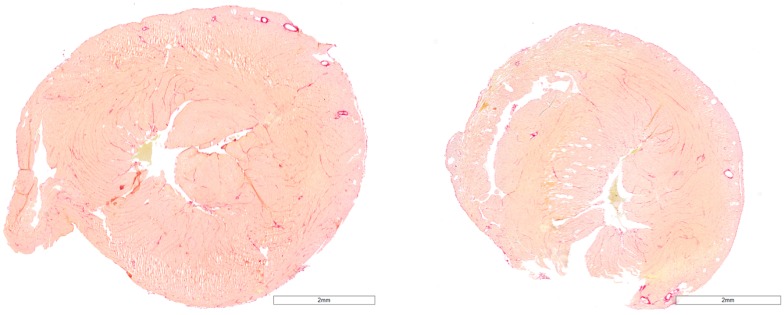
Picro-Sirius red staining for fibrosis assessment of 6 μm myocardial slices from the basal third part of the heart of the mice from group C (control, left panel) and group D (diabetes, right panel).

Fibrosis assessment was performed in 3 mice from group C and 6 mice from group D and revealed values of 7.3±1.4 and 10.3±3.9%, respectively, with no significant difference between groups (Fig 3). Fibrosis negatively correlated with Ecc at w4 in pooled data from groups C and D (r = -0.778; p<0.05).

## Discussion

This study confirmed the early occurrence of diastolic dysfunction in STZ-induced diabetic animals and further demonstrated a very early decrease in left ventricular volumes within one week after diabetes onset, mimicking a restrictive cardiomyopathy.

Previous preclinical rodent studies explored the effect of diabetes on cardiac morphology and function with varying results depending on the measurement methods. Several authors reported systolic and/or diastolic dysfunction and abnormal pressure-volume patterns using echocardiography or ex vivo isolated hearts, which suggests increased left ventricular wall stiffness [17–20]. The primary limitation of these findings is the lack of physiological information that is obtained from ex vivo experiments. CMR has emerged as a powerful tool for *in vivo* assessments of cardiac function in humans and experimental animal models of cardiovascular diseases [21]. DCM has been increasingly explored using CMR in rodent models over the past decade and has demonstrated structural and functional cardiac changes 1 to 2 months after the induction of diabetes [22,23]. We confirmed these findings by showing an early onset of diastolic dysfunction 1 week after STZ injection in our study. In contrast to prior reports and to our study, Yu et al. also found significant thinning of the LV wall in diabetic mice with a normal EDV and increased ESV. Notably, left ventricular morphological and functional measurements were not indexed to body weight in this study, but a 13% weight decrease was observed in diabetic animals, which introduces a potential bias in the conclusions [23]. In our study, we indexed the CMR measurements to body weight where appropriate because diabetic animals presented with a rapid 10% body weight variation in a few weeks. We also used 16-frame gating for functional assessment rather than the low temporal resolution (10-frame gating) used in previous studies [22,23]; our resolution increases the reliability of ESV measurement [24].

Our study explored cardiac outcome within one week after the onset of diabetes and demonstrated an acute decrease in cardiac volumes (EDV, ESV and SV). We hypothesized that this dramatic and early effect was linked to hypovolemia related to hyperglycemic osmotic diuresis. Hyperglycemic osmotic diuresis is observed when the glomerular filtration of glucose exceeds the tubular reabsorption threshold. The osmotic properties of glucose in the tubular lumen increase sodium and water clearance, possibly leading to hypovolemia when this phenomenon is intense. Several results in our study suggest this mechanism. First, the 24-h diuresis was significantly increased in diabetic animals compared to control and furosemide-induced hypovolemic animals. Second, the primary CMR abnormality at week 1 was a decrease in the left ventricular volumes, which contrasts with a preserved left ventricular ejection fraction and normal myocardial strain. This condition is suggestive of a reduced preload. T2 relaxation times, which are related to the amount of myocardial fibrosis [25,26], were also not different between the two groups. This result suggests that collagen deposition between myofibers, which is the most common histological lesion in diabetic cardiomyopathy [27], had not yet begun after one week of hyperglycemia. Later, at week 4, our histological results revealed only a slight increase in myocardial fibrosis in diabetic animals, with a negative correlation between fibrosis and circumferential strain. These two findings are likely related to the ongoing fibrotic process several weeks after diabetes onset, as recently shown in another mouse study [28]. This fibrosis process is a potential pathophysiological pathway between tissue damage and ventricular dysfunction after several weeks of diabetes but is unlikely to have a causal role in the early cardiac volume impairment observed in our study.

The furosemide-induced hypovolemic model was used to support our hypothesis that hypovolemia underlies the early CMR abnormalities in diabetic animals. Furosemide inhibits the sodium-potassium-chloride co-transporter in the apical membrane of tubular epithelial cells in the thick ascending limb, resulting in an increase in urinary sodium and water excretion [29]. Furosemide injections resulted in an acute increase in the 24-h diuresis and profound hypovolemia with dramatically decreased left ventricular volumes, as well as increased biological markers of hypovolemia/dehydration and hypotension. In diabetic animals, diuresis correlated with the glucose level and was even more increased than in animals treated by furosemide. This polyuria resulted in an intermediate phenotype with a mild ventricular volume decrease and a mild plasma aldosterone increase. This mild hypovolemic phenotype in diabetic animals suggests that in the setting of chronic osmotic polyuria, adaptive mechanisms were partially effective, as indicated by the normal blood pressure in this group. Hyperaldosteronemia in hyperglycemic animals is particularly interesting in this situation because this hormone is usually decreased in type 1 diabetes [30]. The aldosterone increase in these animals suggests activation of the renin-angiotensin-aldosterone system in response to hypovolemia. This insight is also reinforced by the positive correlation between blood glucose and plasma aldosterone in diabetic animals. One might hypothesize that hypovolemia might be related to a direct toxic effect of STZ in the kidney, but Palm *et al*. demonstrated that most functional renal disturbances occurring in the STZ-diabetic rodent model reflect the diabetic condition [31]. Another potential bias in our results is the use of insulin in the sole diabetic group, given the demonstrated cardiovascular effect and suspected sodium-retaining actions of this hormone. Insulin has opposing vasodilator and vasoconstrictor actions, and the sodium-retaining effect is offset by compensatory natriuresis, such that the net systemic hemodynamic effect seems minimal [32,33]. However, it would have been interesting to evaluate non-diabetic animals that also received insulin, but the hypoglycemic risk and its cardiovascular consequences (mainly mediated by sympathetic system activation) were considered even more confusing [34].

Overall, all of these elements advocate for an impairment of cardiac preload rather than intrinsic myocardial dysfunction to explain the early left ventricular volume decrease in our hyperglycemic rodent model. This mechanism could partly explain the discrepancy that was observed in a previous comparison of two rodent models of diabetic cardiomyopathy using a pressure-volume catheter [35]. The authors demonstrated that SV was only reduced in STZ-induced diabetic rats with a blood glucose above 20 mmol/L. Conversely, lean Zucker rats, a type 2 diabetes model, presented with mild hyperglycemia and normal SV.

One might even question the relevance of STZ-induced diabetes as a rodent model for DCM assessment because of the potential confusing factors that may affect the evaluation parameters. First, previous reports demonstrated that 20 weeks of STZ-induced diabetes in rats led to a shift to a more compliant isoform of titin, a sarcomeric protein involved in myocardial stiffness [36,37]. Second, STZ-induced diabetes is usually associated, after a few weeks, with a reduced heart rate in rodents, probably via carnitine deficiency [38]. Such relative bradycardia was not observed in our study and was probably thwarted by the hemodynamic adaptation to hypovolemia. Finally, our findings suggest that this STZ-induced diabetes model may lead to very early hypovolemia secondary to hyperglycemia, only one week after STZ injection. These findings are probably specific to this STZ-induced rodent diabetes model, which has a very fast onset for the blood glucose increase. The hemodynamic effects of hyperglycemia in other rodent diabetes models could be different and remain to be explored. Thus, whether through a change in myocardial compliance, a chronotropic effect or volemia impairment via major hyperglycemia, the biases that may interfere with the evaluation of this model are numerous and should be considered in the framework of DCM.

There is a paucity of data on the CMR assessment of diabetic cardiomyopathy in humans. One study explored type 2 diabetic patients and demonstrated lower left ventricular end diastolic volumes in this population compared to normal subjects. This finding was observed in the setting of chronic type 2 diabetes and was also associated with PFR impairment, which suggests myocardial relaxation dysfunction rather than hemodynamic trouble [39].

There is no available data on the potential effect of acute hyperglycemic osmotic diuresis on left ventricular volumes. However, the hypotensive side effects of sodium-glucose cotransporter 2 (SGLT-2) inhibitors, new oral hypoglycemic agents that lower the glucose tubular reabsorption threshold and lead to major glycosuria, suggest a potential role of such glycosuria on cardiac preload [40]. In addition, the recent EMPA-REG study demonstrated a significant reduction in hospitalization for heart failure in patients with type 2 diabetes treated with empagliflozin (an SGLT-2 inhibitor); this benefit occurred from the third month of treatment and continued until 3 years [41]. Furthermore, this improvement is probably multidimensional; we speculate that its early onset might involve glycosuria and subsequent hemodynamic modifications, as described in our study.

## Conclusions

We demonstrated that the early detection of reduced left ventricular volumes in a rodent model of STZ-induced diabetic cardiomyopathy might be explained by the hypovolemia that is secondary to hyperglycemic osmotic diuresis. Future research using diabetic animal models should consider that the observed cardiac alterations might be partially related to hemodynamic changes secondary to hyperglycemia. The relevance of this finding to humans should be explored in future studies.

## Supporting Information

S1 Table Minimal individual data set. (XLSX)
